# Speckle-tracking echocardiography of left and right ventricle and acute cellular rejection in orthotropic heart transplantation: a systematic review and meta-analysis

**DOI:** 10.1007/s10554-024-03297-3

**Published:** 2024-12-05

**Authors:** Eleni Xourgia, Kristina Brignoli, Olivier Linder, Alexandra-Maria Neagoe, Lukas Capek, Jolie Bruno, Eva Strickler, Adam Bakula, Maryam Pavlicek-Bahlo, Monika Fürholz, Christian Muster, Patrizia Malagutti, Michele Martinelli, Lukas Hunziker, Bruno Schnegg

**Affiliations:** 1 Department of Cardiology, Inselspital, Bern University Hospital, University of Bern, Bern, Switzerland; 2 Department of Internal Medicine, Bürgerspital Solothurn, Solothurn, Switzerland

**Keywords:** Cardiac transplantation, Acute cellular rejection, Endomyocardial biopsy, Echocardiography, Speckle-tracking, Strain

## Abstract

After a cardiac transplantation, the steering of immunosuppression requires an active search for acute cellular rejection (ACR). Surveillance with endomyocardial biopsy (EMB) is the gold standard. Given the costs and potential complications, there is growing interest in the use of non-invasive screening methods. Thus, we have conducted a systematic review and meta-analysis to evaluate the role of speckle-tracking echocardiography as a screening method for ACR. We searched PubMed (CENTRAL) and gray literature for studies presenting data on speckle tracking echocardiography in heart-transplant patients experiencing acute cellular rejection. The primary outcomes of the meta-analysis were left and right ventricular global longitudinal strain. We used random effects models for all our calculations. We pre-registered our meta-analysis with PROSPERO (CRD42024508654). By incorporating data from over 2000 biopsies included in 18 studies, we found that both left (LVGLS, MD -1.96, 95% CI -2.85 to -1.07, *p* < 0.0001), and right (RVGLS, MD -2.90, 95% CI -4.03 to -1.76, *p* < 0.00001) ventricular longitudinal strain were lower among patients without ACR. The change of LVGLS from baseline over time was also greater among patients experiencing ACR (MD -2.43, 95% CI -4.82 to -0.05, *p* = 0.045). Current data suggest that myocardial strain measured by speckle tracking echocardiography is affected in ACR and could potentially be used for early rejection detection as a rule-out strategy, leading to reduction of routine EMB in heart transplant follow-up.

**Supplementary information:**

The online version contains supplementary material available at 10.1007/s10554-024-03297-3.

## Introduction

Cardiac transplantation (HTX) is the preferred therapeutic option for individuals afflicted with end-stage heart failure, substituting a failing heart with a viable organ procured from a donor. But this treatment exposes the recipient to the surface cell marker of the donor organ, which in turn activates the recipient’s immune system. To counter the risk of organ rejection, patients must be prescribed immunosuppressive (IS) therapy. To balance the risk of infection and rejection, fine-tuning of the IS dose is required. This is typically achieved through right ventricular endomyocardial biopsies (EMB’s). This procedure is invasive and carries a complication rate of approximately 6% [1].

In recent years, less invasive alternatives as emerged for rejection screening, such as gene expression profiling (GEP) or donor-derived cell-free DNA (dd-cfDNA) who appear to be a promising alternatives to EMB’s [2–5]. Extracellular volume and T2-Mapping in magnetic resonance imaging has also been examined as a potential alternative for rejection diagnosis, with the added advantage of cardiac imaging being more widely available in comparison to the aforementioned peripheral blood testing [6]. Unfortunately, these techniques remain insufficiently specific and difficult to implement. Hence, official recommendations still limit their use to low-risk adult patients [7].

On the other hand, the use of echocardiography is simple, accessible and inexpensive. Unfortunately, the use of the traditional echocardiographic parameters, with measurement of left ventricular function and variation in ventricular wall thickness (surrogate for cellular oedema), has been disappointing in detecting asymptomatic rejection [8]. Nonetheless, more advanced measurements such as myocardial deformation have been shown to detect ventricular systolic impairment before a decrease in ejection fraction (EF) occurs in various myocardial dysfunction settings [9].

Specifically in acute cellular rejection (ACR), myocardial strain measured by tissue Doppler imaging has been shown to be sensitive in detecting asymptomatic rejection [10]. The first meta-analyses on the subject showed promising results for left ventricular global longitudinal strain, circumferential strain and right-ventricular free-wall strain, albeit, at the time, with much fewer patients and no data presented on right ventricular global measurements [11, 12].

However, tissue Doppler imaging strain measurements has subpar reproducibility rates due to being angle-dependent and needing to be positioned parallel to left-ventricular wall motion, a technical restraint that is overcome by speckle-tracking echocardiography (STE) [13].

Thus, we aimed to conduct a systematic review and meta-analysis of all published data examining the difference in STE-measured strain of left and right ventricle in both ACR and non-ACR heart transplant patients. We accumulated the emerging data of the last years and discussed the potential of STE in post-transplant follow-up.

## Methods

### Protocol and registration

The current systematic review and meta-analysis is reported in accordance with the Preferred Reporting Items for Systematic Reviews and Meta-Analyses (PRISMA) statement (Supplementary Appendix, Table S1). We pre-registered the protocol with PROSPERO (CRD4202450865) and made it available online.

### Inclusion and exclusion criteria

We considered for inclusion studies reporting presenting data on acute cellular rejection on heart transplant patients with concurrent data on speckle tracking echocardiography. We considered as eligible both peer-reviewed papers and preprints, while we excluded case reports and case series involving less than five patients.

### Outcomes of interest

The primary outcomes were to investigate the difference in left and right ventricular global longitudinal strain between non-ACR and ACR patients. ACR was defined according to the ISHLT classification as ≥ 2R in all included studies, expect from the study of Ciarka et al. in which the ACR group included only ≥ 3R (severe rejection) patients versus healthy controls. Secondary outcomes included the left ventricular circumferential and radial strain (LVCS, LVRS), the right ventricular free-wall strain (RVFWS) and the left- and right- ventricular strain rates (longitudinal strain rate, LSR, circumferential strain rate, CSR).

### Search strategy

Three authors (EX, KB and OL) independently conducted the literature search. We systematically searched PubMed (CENTRAL) in order to explore all available clinical studies on the topic with the search phrase: (“strain” OR “speckle”) AND (“transplant*”) AND (“rejection”). We also conducted a search in the grey literature (i.e., preprint servers, medRxiv and Research Square and Google Scholar) by using the same search phrase. Finally, a “snowballing” search was conducted in the reference lists of all identified studies. We retrieved all relevant articles up to January 31st 2024, with no language restrictions.

### Data extraction

The titles and abstracts of studies obtained using the search strategy and those from additional sources were independently screened by three authors (EX, KB and OL) to identify studies that potentially meet the inclusion criteria outlined above. The data from each study were independently extracted by three authors (EX, KB and OL).

A pre-designed form was used to extract data from the included studies. Extracted information included publication and study details (authors, year, country), cohort characteristics (patients in each group, definition of rejection, echocardiographic measurements) and outcomes.

When not directly provided, we calculated data of interest, i.e., by transforming continuous values to mean and standard deviation as described by the Cochrane Handbook version 6.4, 2023. Discrepancies were resolved through discussion or with the input of the other authors if necessary. We contacted the authors of the original studies for clarifications and/or additional information but did not include any unpublished data in our review.

### Risk of bias assessment

Articles included in the analysis were independently assessed by four authors (EX, KB, OL and AMN) for methodological quality before inclusion in the review using the ROBINS-I tool for non-randomized studies of interventions as proposed in the Cochrane Handbook version 6.4, 2023.

### Statistical, sensitivity and subgroup analyses

Data synthesis for the double-arm trials was conducted using Review Manager 5.4 (RevMan 5.4.1) by the Cochrane Collaboration. Continuous effect measures were pooled as mean difference (MD) with 95% confidence intervals (CI). Continuous values were transformed and presented as medians to means, and interquartile range (IQR) to standard deviation (SD) as instructed by the Cochrane Handbook. A random effects model was conservatively utilized. A p-value less than 0.05 was considered to denote statistical significance. The metafor and nlme packages of R were used for the pre/post-effect size meta-analysis including both baseline and follow-up data according to the models proposed by Papadimitropoulou et al. [14]. The presence of statistical heterogeneity was assessed by *I*^2^ and interpreted according to the Cochrane Handbook recommendations. To explore sources of heterogeneity for our primary outcomes, a pre-specified sensitivity analysis was conducted by including only studies with low risk of bias and a pre-specified subgroup analysis was performed in studies with patients exclusively in the first year of follow-up versus cohorts including patients at various follow-up time points. We also visually inspected the funnel plots and conducted an Egger’s test to test for the risk of small study effect and publication bias on the analysis of all our main outcomes. A leave-one-out analysis for both main outcomes was performed to explore heterogeneity. Results of the final analyses are visually presented as forest plots. All data used in the review are available upon request.

### Strain terminology

In order to avoid unclear terminology given the fact that strain is reported in negative numbers, “increased strain” in our study will be referring to less negative numbers (i.e. absolute smaller numbers signifying worse myocardial function).

## Results

**Fig. 1:**
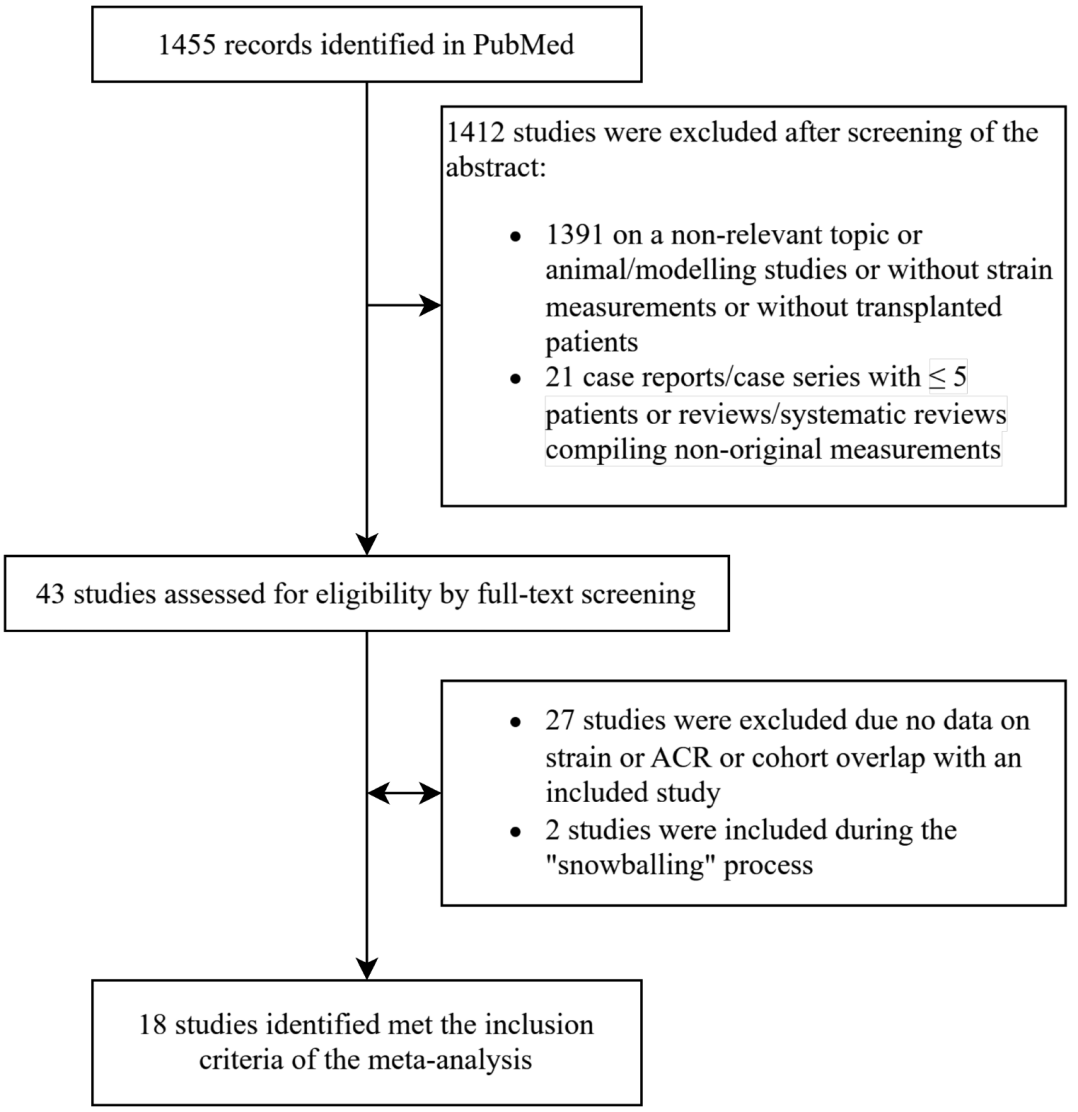
Study flow chart

Of the 1455 relevant citations that were identified and screened, 43 studies were selected for full review based on their abstract and were included in our final assessment for possible data extraction (Fig. 1). In total, data extraction was feasible in 18 studies including a total of 915 patients with over 2000 endomyocardial biopsies [10, 15–31]. One study [32] was excluded due to overlap with another included study [20] from the same author group. Additionally, overlap existed also between two other cohorts [25, 28] with the one with the smaller patient population being excluded from the analyses of all outcomes except radial and circumferential strain for which the larger cohort presented no data. Finally, Antończyk et al. presented overlapping data on two publications, with one being included in the primary analysis [23] and one in the strain over time analysis [33]. Table 1 depicts the baseline characteristics of the transplanted patient populations in the studies included in the meta-analysis. Results regarding risk of bias assessment of included studies are summarized in the supplementary appendix (Table S2).

**Table 1:** Baseline characteristics of included studies^a, b^

PMID	Study name	Year	Country	Type of study	Echocardiography hardware and software	Patients (EMBs), *n*	Age, y	Sex (male), %	Follow-up period after HTx
20,947,044	Eleid et al.	2010	USA	Prospective	Acuson Sequoia (Siemens Medical Solutions USA, Mountain View, California), Vivid 7 (GE Healthcare, Milwaukee, USA) and vendor-customized 2D Cardiac Performance Analysis software (TomTec Imaging Systems, Munich, Germany)	31 (N/A)	52.6 ± 12.9	75	2 years
24,814,366	Ruiz Ortiz et al.	2015	Spain	Prospective	iE33 (Philips Medical Systems, Amsterdam, The Netherlands) and QLab (Philips Medical Systems)	20 (78)	51.0 ± 15.0	80	1 year
26,165,446	Mingo-Santos et al.	2015	Spain	Prospective	iE33 (Philips Medical Systems, Best, The Netherlands) and X-Celera (Philips Medical Systems)	34 (235)	51.8 ± 12.3	82	1 year
25,942,427	Podrouzkova et al.	2015	Czech Republic	Prospective	Vivid E7 (GE Healthcare, Milwaukee, USA) and EchoPAC (GE Healthcare)	43 (N/A)	51.3 ± 3.6	67	11 days to 4 years
25,622,999	Ambardekar et al.	2015	USA	Retrospective	Device N/A and Velocity Vector Imaging (Siemens Medical Systems, Mountain View, CA)	44 (N/A)	44.4 ± 13.8	77	1 year
25,499,656	Clemmensen et al.	2015	Denmark	Retrospective	Vivid E9 (GE Vingmed Ultrasound AS, Horten, Norway) and EchoPAC (GE Vingmed Ultrasound AS)	64 (509)	45.8 ± 13.5	67	N/A
28,295,946	Wisotzkey et al.	2017	USA	Retrospective	iE33 (Philips Healthcare, Andover, MA, USA) and Philips QLAB (Philips Medical Systems, Best, The Netherlands)	47 (N/A)	9.1 ± 6.5	55	3.4 ± 4.1 years
30,579,333	Tseng et al.	2018	USA	Retrospective	Vivid E9 (GE Healthcare) and Velocity Vector Imaging (Siemens Medical Solutions USA, Inc)	65 (N/A)	51.3 ± 12.4	71	1.0 ± 0.8 years
30,177,115	Antończyk et al.	2018	Poland	Prospective	Vivid E9 Ultrasound System (GE Healthcare) and EchoPAC (GE Healthcare)	45 (220)	49.5 ± 11.4	80	1 year
29,680,337	Sade et al.	2019	Turkey	Retrospective	Acuson SC 2000 (Siemens Medical Solutions USA, Mountain View, CA) and Velocity Vector Imaging (Siemens Healthcare, Erlangen, Germany)	31 (N/A)	41.5 ± 15.5	67	N/A
32,297,099	Ruiz Ortiz et al.^c^	2020	Spain	Prospective	Acuson SC 2000 (Siemens AG, Erlangen, Germany) and Velocity Vector Imaging (Siemens AG, Erlangen, Germany)	37 (251)	52.0 ± 14.0	62	1 year
32,074,202	Carrion et al.	2020	Brazil	Retrospective	Device N/A and TomTec (TomTec Imaging Systems, Unterschleißheim, Germany)	19 (170)	48.0 ± 12.4	42	18 months
33,422,079	Cruz et al.	2021	Brazil	Prospective	Vivid E9 Ultrasound System (GE Healthcare) and EchoPAC (GE Healthcare)	60 (60)	42.1 ± 11.5	57	6 months
32,205,100	Artaza et al.^c^	2021	Spain	Prospective	iE33 (Philips Medical Systems, Best, The Netherlands) and QLab (Philips Medical Systems)	99 (501)	58.0 ± 11.8	79	1 year
34,687,539	Ciarka et al.	2022	Belgium	Retrospective	Device N/A and EchoPAC (GE Vingmed Ultrasound, Horten, Norway)	27 (N/A)	42.9 ± 16.5	86	13.0 ± 23 months
36,434,347	Chamberlain et al.	2022	Australia	Prospective	iE33 (Philips, Andover, MA, USA) and Vivid E9 (GE Medical, Milwaukee, WI, USA) and Image arena (TomTec imaging systems, Unterschleißheim, Germany)	128 (N/A)	47.8 ± 15.3	82	1 year
36,312,230	Costa et al.	2022	Brazil	Prospective	EPIQ 7 and EPIQ CVx (Philips, Koninklijke, Netherlands) and TomTec (TomTec Imaging Systems, Unterschleißheim, Germany)	54 (105)	50.3 ± 10.9	60	1 year
35,571,160	Otto et al.	2022	Brazil	Prospective	Vivid E9 (GE Healthcare, Milwaukee, USA) and EchoPAC (GE Vingmed Ultrasound, Norway).	67 (67)	52.0 ± 10.6	40	1 year

Abbreviations: EMBs, endomyocardial biopsies; n, number; y, year; HTx, heart transplantation; N/A, not available

^a^If the original data was not provided, it was calculated or converted (e.g. median and interquartile range to mean and standard deviation)

^b^Acute cellular rejection was defined according to International Society for Heart and Lung Transplant criteria as ≥ 2R in the majority of included cohorts, in cohorts were light rejection (1R) data was provided separately, those data were merged with the 0R cohort into a “no rejection” group for the purposes of the analyses. Exceptions were the cohort of Ciarka et al., where the rejection cohort was defined as ≥ 3R and the cohort of Chamberlain et al., that had a moderate rejection group with one episode of ≥ 2R ACR or > 50% of biopsies with 1R, that was merged with the severe rejection group for the purposes of the analyses

^c^There is definite overlap between the two cohorts; data from Artaza et al. were used for the majority of analyses given the larger cohort size, data from Ruiz-Ortiz et al. were used for the analyses of circumferential and radial strain

### Primary outcomes – left and right ventricular global longitudinal strain

**Fig. 2:**
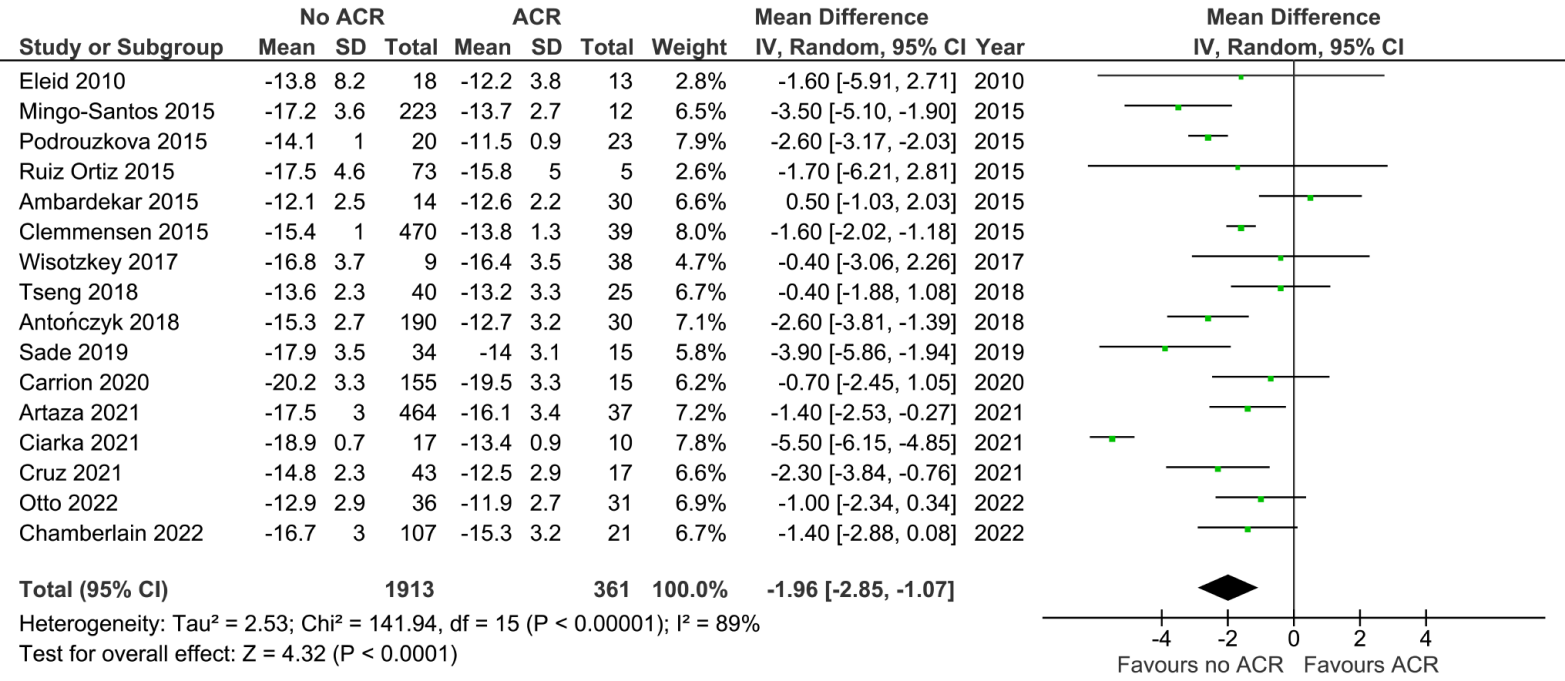
Left ventricular global longitudinal strain in ACR versus non-ACR controls. Mean difference (MD) and 95% confidence intervals (CI) were calculated using a random effects model

**Fig. 3:**
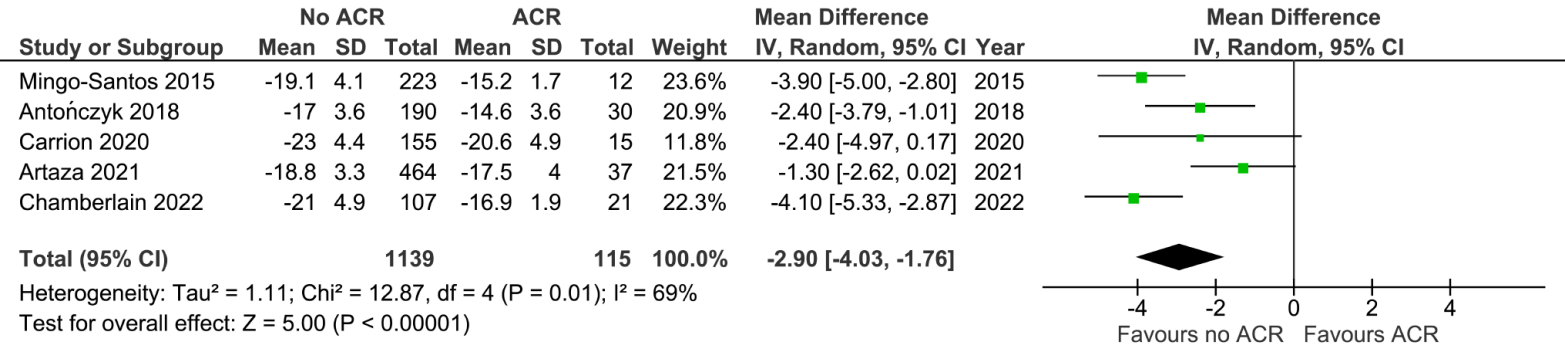
Right ventricular global longitudinal strain in ACR versus non-ACR controls. Mean difference (MD) and 95% confidence intervals (CI) were calculated using a random effects model

Figure 2 shows that LVGLS was increased in ACR versus non-ACR controls (MD -1.96, 95% CI -2.85 to -1.07, *p* < 0.0001; 16 studies; 2274 EMBs). Figure 3 similarly shows that RVGLS was increased in ACR versus non-ACR controls (MD -2.90, 95% CI -4.03 to -1.76, *p* < 0.00001; 5 studies; 1254 EMBs). For LVGLS the Egger’s test for publication bias yielded a p-value of 0.279 and an intercept of -1.26. For RVGLS the Egger’s test produced a p-value of 0.459 and an intercept of -0.95.

The results persisted in the sensitivity analysis including only 10 low risk of bias cohorts out of the initial 16 included studies for left (MD -1.92, 95% CI -2.45 to -1.39, *p* < 0.00001; 1930 patients) and 4 low risk of bias cohorts out of the initial 5 included for right (MD -2.55, 95% CI -3.84 to -1.27, *p* = 0.0001; 1126 EMBs) ventricular measurements.

To explore the high heterogeneity in our main outcome analysis, we performed a leave-one-out analysis for both LVGLS and RVGLS, that did not identify any studies that acted as outliers and thus influenced the main result.

### Secondary outcomes – left ventricular circumferential and radial strain, strain rates and right ventricular free-wall strain

**Fig. 4:**
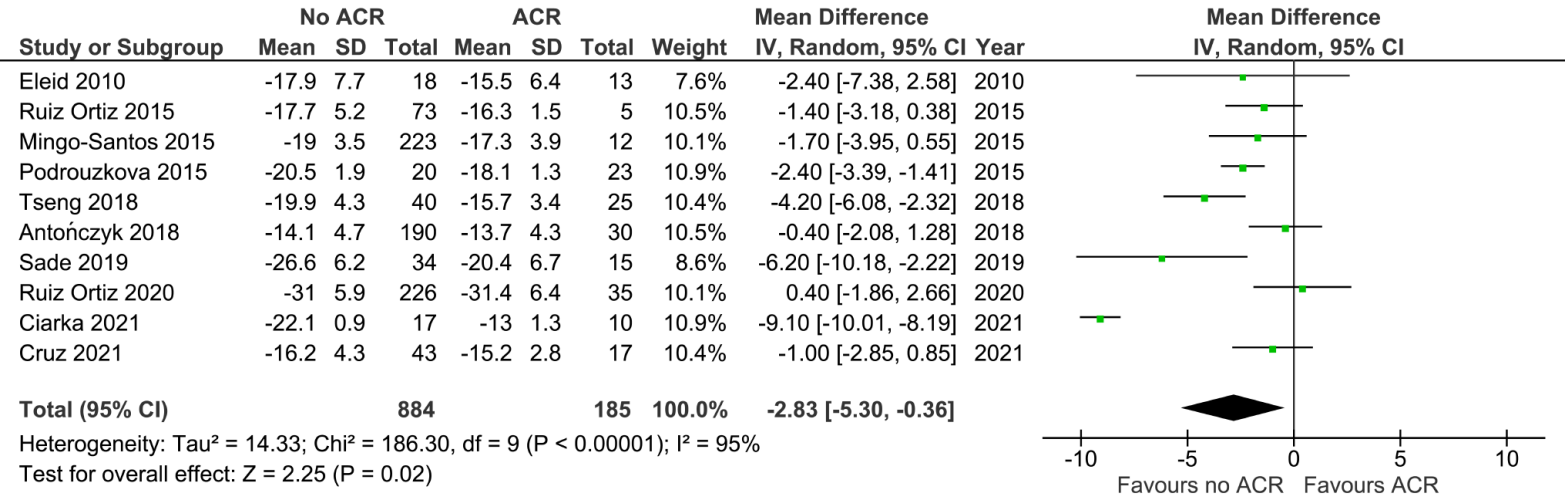
Left circumferential strain in ACR versus non-ACR controls. Mean difference (MD) and 95% confidence intervals (CI) were calculated using a random effects model

**Fig. 5:**
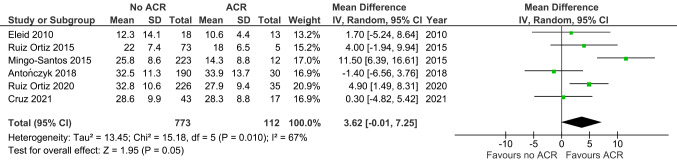
Left radial strain in ACR versus non-ACR controls. Mean difference (MD) and 95% confidence intervals (CI) were calculated using a random effects model

Similar to longitudinal strain, LVCS (MD -2.83, 95% CI -5.30 to -0.36, *p* = 0.02; 10 studies; 1069 EMBs; Fig. 4) was increased in ACR versus non-ACR controls. LVRS (MD 3.62, 95% CI -0.01 to 7.25, *p* = 0.05; 6 studies; 885 EMBs; Fig. 5) was similar between the two groups.

**Fig. 6:**
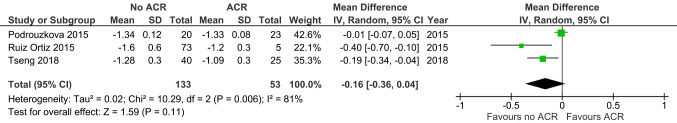
Circumferential strain rate in ACR versus non-ACR controls. Mean difference (MD) and 95% confidence intervals (CI) were calculated using a random effects model

**Fig. 7:**
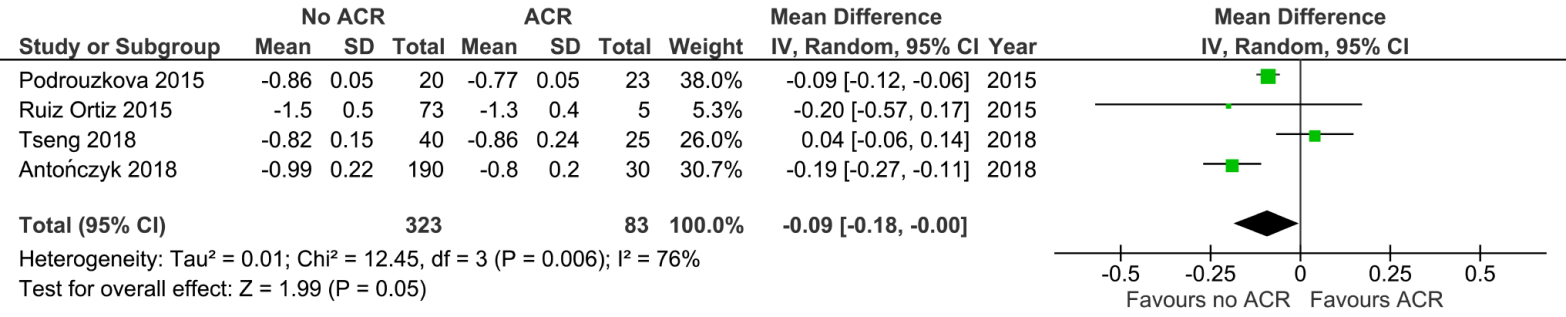
Longitudinal strain rate in ACR versus non-ACR controls. Mean difference (MD) and 95% confidence intervals (CI) were calculated using a random effects model

Both circumferential (MD -0.16, 95% CI -0.36 to 0.04, *p* = 0.11; 3 studies; 186 EMBs; Fig. 6) and longitudinal strain rates (MD -0.09, 95% CI -0.18 to -0.00, *p* = 0.05; 4 studies; 406 EMBs; Fig. 7) were similar between groups.

**Fig. 8:**
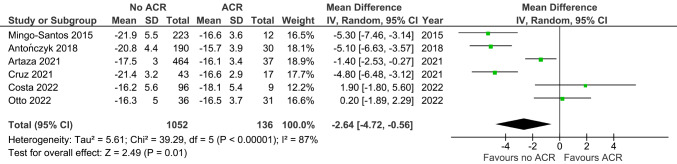
Right ventricular free-wall strain in ACR versus non-ACR controls. Mean difference (MD) and 95% confidence intervals (CI) were calculated using a random effects model

RVFWS was increased in ACR versus non-ACR controls (MD -2.64, 95% CI -4.72 to -0.56, *p* = 0.01; 6 studies; 1188 EMBs; Fig. 8).

### Subgroup analysis – first year after transplant versus various follow-up times

In the subgroup analysis of cohorts with patients strictly in the first year after HTx versus various follow-up times, LVGLS was persistently increased in the ACR cohorts in both groups, with no between-group difference observed (Chi^2^ = 0.44, *p* = 0.51).

### Pre-post effect size meta-analysis – between group differences in strain from baseline to follow-up

In addition to our other analyses, we performed a non-prespecified analysis comparing a “final-score” LVGLS between the two groups that take into account both strain at the time of rejection (or follow-up, for the control group), as well as the baseline value for each group in an ANCOVA approach. In this analysis, the change of LVGLS from baseline to follow-up was also greater among patients experiencing rejection (MD -2.59, 95% CI -4.82 to -0.35, *p* = 0.02; 4 studies; 145 EMBs).

## Discussion

This systematic review and meta-analysis accumulating data from 18 studies, which included a total of 915 patients and over 2000 endomyocardial biopsies (which 361 diagnosed an acute rejection) found that LVGLS, RVGLS, LVCS and RVFWS were increased in clinically relevant acute cellular rejection compared to controls, while there was no difference in LVRS and circumferential or longitudinal strain rates. In the subgroup analysis examining patients in the first year of follow-up versus all others, LVGLS was increased in rejection irrespective of when it occurred during the follow-up. Those findings along with the very high intra- and interobserver reproducibility of measurements in the original cohorts, support that STE could be used for non-invasive ACR screening in the post-transplant setting, both in the first year after transplant and in long-term follow-up.

It should be noted that despite our pooled data supporting that LVCS is also affected in ACR episodes, many of the individual studies have failed to show a difference between patients and controls. The authors of the primary cohorts have attributed the lack of difference of LVCS to the early stages of edema or fibrosis affecting subendocardial myocardial muscle fibers, which leads to isolated decrease of longitudinal function [23]. The aforementioned theory along with the fact that the study of Ciarka et al. included ≥ 3R patients in the rejection cohort (while in other studies clinically-relevant rejection was defined as ≥ 2R) could explain why this cohort is an outlier in our analysis, with LVCS being more heavily affected in rejection in this study [29]. Thus, in suspected cases of light rejection this metric may not be suitable as a single measurement and should be better evaluated in conjunction with other strain values.

### Strain over time and single-point cut-off values

The main limitation for the use of STE as a screening tool appears to be the lack of a validated cut-off for detecting ACR. Especially in the first post-transplant year, the cut-off values validated in general population cannot be applied given the observation that longitudinal dysfunction with compensatory changes in circumferential strain occur in that time period irrespective of the presence of rejection [34]. A worsening of longitudinal function in this early phase could be an indicator of pathological myocardial dysfunction owing to rejection. Accordingly, in our analysis, the increase of LVGLS from post-transplant baseline until the rejection-event (or follow-up for non-rejection patients) was greater in ACR cases, indicating that evaluating the strain trajectory could also be useful in early rejection detection without being limited by the disadvantages of single-point measurements.

As mentioned above, the lack of validated cut-off values for single-point strain measurements is the largest drawback of using STE in post-transplant surveillance. Only few of the primary studies included in our systematic review have provided cut-off values for single or combined measurements that can detect ACR with high sensitivity and specificity [17, 20, 23, 27]. Those are presented in detail in Table 2. The proposed cut-offs derived from study-specific optimal points in the receiver-operator curve for diagnosing rejection and were not at the time of publication validated in external cohorts. Among those, the highest sensitivity was achieved with the cut-offs of GLS < 14% and GCS ≤ 24% and a combination of LVGLS < 15.5% and RVFWS < 17%, while the most specific combination was RVFWS ≤ 16.8% and 4-CH-LS ≤ 13.8% [17, 23, 24]. The negative predictive value of strain measurements was universally very high underscoring the utility of strain in ruling out suspect cases of ACR and thus minimizing unnecessary EMB burden.

**Table 2:** Cut-off values of myocardial strain for detecting acute cellular rejection^a^

	Study name	Proposed cut-off value (sensitivity/specificity/negative predictive value, %)
• 26,165,446	Mingo-Santos et al.	• RVFWS < 17% (85.7/91.1/98.8) • LVGLS < 15.5% (85.7/81.4/98.8) • LVGLS < 15.5% and RVFWS < 17% (100/77.0/100)
• 25,499,656	Clemmensen et al.	• GLS ≤ 13.5% (55.6/75.0)
• 30,177,115	Antończyk et al.	• RVFWS ≤ 16.8% (73/82/95) • 4-CH-LS ≤ 13.8% (87/72/97) • RVFWS ≤ 16.8% and 4-CH-LS ≤ 13.8% (63/93/94) • GLS ≤ 14.1% (77/72/95)
• 29,680,337	Sade et al.	• GLS < 14% (100/83/100) • GCS ≤ 24% (100/62/100)
33,422,079	Cruz et al.	• RV-FWLS < 18% and troponin > 0.05 ng/mL had AUC 0.89 with 95%CI:0.81–0.93

Abbreviations: RVFWS, right ventricular free-wall strain; LVGLS, left ventricular global longitudinal strain; GLS, global longitudinal strain; 4-CH-LS, four chamber longitudinal strain; AUC, area under the curve; CI, confidence interval

^a^All strain values are presented as absolute numbers

### Future aspects

While current studies compare STE in ACR versus non-rejection controls, no randomized controlled trials directly comparing STE to EMB for ACR detection have been published yet.

Given the very high negative predictive values of strain measurements in existing clinical trials, the use of STE could be used for screening (as rule-out). Suspected cases would then be submitted to the EMB to confirm or refute the suspicion of rejection. This strategy could be compared in a randomized fashion to a group of patients following the current standard protocol (serial biopsy at given intervals).

In addition to echocardiography other widely available non-invasive methods are also being increasingly validated for ACR detection. The high sensitivity and negative predictive value of dd-cfDNA and GEP has been shown in multiple trials, also in direct comparison to EMB [3, 4, 35] leading to their implementation in real-world surveillance protocols [36] and further ongoing randomized controlled-trials in order for definitive conclusions to be drawn (NCT05081739). Lower cost alternatives are also being tested such as in the recent study of Adedinsewo et al., where a deep learning model was trained with electrocardiogram and biopsy pairs in order to be able to detect ACR from 12-lead electrocardiogram recordings [37].

Similarly, other non-invasive imaging modalities such as cardiac magnetic imaging have also been showing high diagnostic performance (area under the curve 0.92 for multiparametric assesment) in detecting ACR and thus reducing the need for EMBs [38].

Whether combining STE with one of those methods is of added benefit in the detection rates of ACR remains to be examined in future studies.

### Limitations

Our meta-analysis has several limitations. Firstly, the retrospective character of several of the included studies increases the risk of bias of the pooled results. To address this limitation, we performed a sensitivity analysis for our primary outcomes including only studies with low risk of bias that confirmed our initial findings. Secondly, our analysis on strain rates was based on a small number of studies and participants, thus calling into question whether the lack of observed effect is true or a result of the analysis being underpowered for detecting a true effect. This could be clarified with publication and subsequent analysis of more data on those metrics in a future meta-analysis. Thirdly, the heterogeneity of our analyses was high, ranging from 67 to 95%, to address this limitation we conducted a leave-one-out analysis for our two main outcomes, which however did not identify a single outlier cohort that could explain this discrepancy. Since in the subgroup analysis of studies with patients in the strictly in the first year after transplant versus patients in various follow-up points the heterogeneity of the later group was very high in comparison to that of the former (94% vs. 59%, respectively), we attributed the observed heterogeneity at least partly to the varying follow-up times and varying graft ages in those cohorts. Furthermore, since cardiac allograft vasculopathy has been shown to affect strain measurements, especially in long-term transplant recipients and its presence was not controlled in our included cohorts, this could be a potential source of heterogeneity and limitation of this diagnostic method. Finally, due to a lack of randomized controlled trials directly comparing EMB to echocardiographic ACR rejection rates, we could not perform a head-to-head comparison of both methods. This limitation cannot be overcome due to the study design of the available published trials and a direct comparison remains to be done in future studies.

## Conclusions

In conclusion, our meta-analysis provides compelling evidence that STE could serve as a valuable non-invasive tool for ACR screening in heart transplant recipients. The significant findings of increased LVGLS, RVGLS, LVCS and RVFWS in our pooled analysis, as well as their high sensitivity and negative predictive value shown in individual studies underscore the potential of STE in the early detection of rejection.

Despite challenges such as the absence of validated cut-off values for strain measurements in the post-transplant setting, our analysis suggests that monitoring strain trajectories over time could be an alternative for the early identification of ACR that overcomes this limitation. Future research targets for further validation of this method include randomized controlled trials comparing STE directly to EMB and the exploration of combining STE with other non-invasive methods to further improve the sensitivity and specificity of ACR detection. Widespread integration of STE into routine post-transplant care could significantly reduce the reliance on EMB, thereby minimizing patient risk and reducing the cost of post-transplant patient management.

## Electronic supplementary material

Below is the link to the electronic supplementary material.

Supplementary Material 1

## Data Availability

No datasets were generated or analysed during the current study.
